# Evaluation of a tactile breath pacer for sleep problems: A mixed method pilot study

**DOI:** 10.3389/fdgth.2022.908159

**Published:** 2022-10-05

**Authors:** Sascha Vermeylen, Elisabeth Honinx, Stefanie Broes, Hilde Vandenhoudt, Nele A. J. De Witte

**Affiliations:** ^1^ LiCalab, Thomas More University of Applied Sciences, Geel, Belgium; ^2^ Moonbird B.V., Brussels, Belgium

**Keywords:** insomnia, sleep problems, breath pacer, breathing exercises, usability, user experience, acceptability, user-centered design

## Abstract

Sleep problems, like insomnia, are a prevalent condition associated with major health risks. Prevention and treatment of sleep problems are thus essential to preserve physical and mental health. Previous work supports the effectiveness of breathing guidance for sleep problems and recommends breathing exercises as an effective intervention for insomnia. While new technologies can support breathing guidance, such novel devices should be assessed for effectiveness and usability to facilitate implementation and continued use. The current pilot study investigates the acceptability and usability of a mobile tactile breathing device and explores its potential impact on subjective sleep quality. In this mixed-method pilot study, 39 participants tested the breathing device for one month in naturalistic circumstances. We collected their experiences, subjective sleep quality, and feedback regarding the usability of the device and the accompanying app through a survey in a pre-post design. The results show that the breathing device is an acceptable solution for sleep problems and participants particularly appreciate the standalone function and design. Nevertheless, important points of attention, such as the size of the device, were also identified. Explorative analyses suggest that subjective sleep quality improved after using the device and accompanying app. The current study supports the usability and acceptability of a tactile breath pacer and provides preliminary evidence supporting a positive impact of the technology on the sleep quality of participants. Recommendations for developers of breathing technologies and eHealth are devised based on the findings.

## Introduction

A good night's sleep is a basic human need. It is important for mental and physical well-being and determines the level of performance during the day (1–3). Sleep disorders are a significant problem in our society whose (economic) consequences are underestimated. An estimated 20%–30% of the general population worldwide experience sleep problems, which can include an inadequate quality, timing, or amount of sleep (4). In Belgium, one in three adults struggle with sleep problems, and reported sleep problems increased over recent years (5, 6). Increasing age, lower socioeconomic status, and being female are independent risk factors (4, 6). The most common sleep disorder is insomnia. This disorder involves difficulty in initiating sleep, maintaining sleep, or waking up too early and not being able to fall asleep again, combined with a feeling of sleepiness and impaired functioning during the day (5, 7). Sleep problems, such as insomnia, often result in daytime distress, lower productivity and compromised quality of life (4). They are also associated with major health risks such as obesity, cardiovascular diseases, diabetes, depression, anxiety, neurodegenerative conditions (8–17), and reduced life expectancy (18, 19). Prevention, early diagnosis, and treatment of sleep problems are thus essential to preserve physical and mental health.

While medication is traditionally considered an easy way to address sleep problems, it is associated with several side effects significantly affecting people's quality of life such as daytime drowsiness and dizziness (20, 21). Furthermore, certain medications can be physically and psychologically addictive (22). Long-term treatment of sleep problems with these medicines can result in dependency and increasing tolerance (23), which can lead to even more side-effects associated with the need to take larger dosages to obtain the desired effect, and physical and mental withdrawal effects (24). Today, overconsumption of prescription medication is reported (25). People suffering from sleep problems as well as physicians and psychologists plead for more non-pharmacological alternatives (26–28). This need has translated into a strong shift towards non-pharmacological solutions such as adopting sleep hygiene measures, e.g., regular exercise, avoiding caffeine and smoking, keeping regular sleeping hours, cognitive behavioral therapy (CBT), and relaxation techniques such as meditation (7, 29). During the last years, breathing exercises have been put forward as a method of choice to cope with sleep problems. In its updated guidelines of 2021, the American Association for Sleep Medicine (AASM) includes and recommends breathing exercises as an effective way to treat chronic insomnia disorder in adults (30).

With breathing techniques, a person consciously and voluntarily changes one or more respiration parameters (e.g., breath pace, breathing depth, or inspiration/expiration ratio). This is different from other relaxation techniques, such as mindfulness or meditation, where a person merely directs his attention to the act of breathing without actually changing it (i.e., breath awareness) (31). Different breathing techniques already exist today. Widely known techniques are the Buteyko method (primarily used for the treatment of asthma) and the Wim Hof method (a combination of fast-paced breathing exercises, training and gradual exposure to cold) (32, 33). In addition, various forms of breathing exercises exist like alternate nostril breathing (alternating between two nostrils), abdominal or diaphragmatic breathing (belly breathing), nasal or oral breathing, or biofeedback breathing (learning to control bio signals like heart rate *via* breathing) (34).

When an individual is breathing at a frequency lower than the spontaneous breathing pace (around 10–20 breaths per minute), this is referred to as slow-paced breathing (35). This is a breathing technique with controlled in- and exhalations, within the range of 4–10 breaths per minute (36). In contrast to uncontrolled fast breathing, generally linked to anxiety and stress, slow-paced breathing has been associated with relaxation and wellbeing (36). Several studies show that practicing slow-paced breathing activates the parasympathetic nervous system (the brake pedal of the body causing the rest-and-digest response) and decelerates the sympathetic nervous system (the gas pedal of the body causing the flight or fight response) (37–43). The interaction between these branches of the nervous system also modulates heart rate. A normal heart rate is irregular, even during resting conditions as a result from complex interactions between different physiological, environmental, and personal determinants, such as breathing rate but also circadian rhythm, temperature, or age (44). The variation in time intervals between successive heartbeats is referred to as heart rate variability (HRV). HRV provides critical information about the functioning of the nervous system and the adaptability of the cardiovascular system. A low HRV has been associated with hypertension, depressive symptoms, anxiety, panic, post-traumatic stress disorder and cardiac mortality (45). In contrast, a high HRV is related to relaxation and reflects the ability to cope with stressful situations (46). Research indicates that externally-paced (or guided) breathing exercises are more effective in inducing relaxation (and a high HRV) than self-paced breathing (47).

In line with this, breath guidance tools have increasingly been developed in recent years. Some of these devices combine breathing guidance with biofeedback to induce relaxation (48). Biofeedback helps to improve performance and health by gaining voluntary control over real-time physiological processes, such as HRV (49, 50). It gives users real-time insights into biosignals, providing a better understanding of the impact breathing has on the body. Today most devices express breathing guidance visually in graphics or numbers, which tends to be rather technical, or *via* auditory cues, which can be cognitively demanding to follow (51). Some devices or apps are also performance-oriented by making use of gamification elements, which can lead to increased stress or anxiety (51). In addition, breath pacers *via* smartphone apps are not always welcomed in an already overly digital world, especially not in a bedroom environment where exposure to exciting stimuli and blue light should maximally be avoided (52, 53). New developments in mHealth and wearable devices allow for the use of other sensory stimuli, like haptic or tactile cues to guide the user, which can help in overcoming these limitations. However, the usability of such breathing devices is often insufficiently assessed.

While the effectiveness of breathing guidance for relaxation and sleep problems has been supported by previous research (46), digital health technologies need to be user-friendly and fit into the context of use of the target population to facilitate adoption and implementation. Today, there is a lack of research into the acceptability, usability, and user experiences of breathing devices. Such research can be executed by living labs, which are open innovation systems that facilitate the evaluation of solutions with end users in simulated or, in this case, realistic circumstances (54).

Therefore, the current pilot study investigates the acceptability and usability of a mobile tactile breathing device and aims to provide preliminary evidence regarding its impact on subjective sleep quality in people with sleep problems. This study specifically aims to examine how and when users interact with the breathing device and assess the usability of the breathing device and accompanying app. Additionally, an exploratory pre-post comparison of subjective sleep quality was conducted to provide preliminary insights into whether breathing exercises provided by a tactile breathing device have the potential to improve sleep quality.

## Materials and methods

### Setting and recruitment

The study was conducted by LiCalab (Living / Care lab), which is a panel-based Belgian living lab specialized in health. Individuals with self-reported sleep problems were invited to participate through e-mails to the panel, newsletters to the employees of Thomas More University of Applied Sciences, and social media posts. Interested individuals applied for the study through an online registration form in which they stated they: (1) suffered from insomnia for at least three months—this was defined as having trouble falling asleep and/or continuing to sleep at least three times a week and, as a result, functioning less well during the day (5); (2) were aged between 18 and 70 years old; (3) were in possession of a smartphone and were able to install an app; (4) were willing to use the tactile breath pacer and to fill in the questionnaires during the test period; (5) were not on holiday for more than three days during the test period (as this might influence sleeping habits). The study aimed to include a population with a diverse age range since user experience can vary depending on age. Inclusion occurred in two waves of 20 participants (due to the availability of devices) and ran between the 15th of June and the 24th of September 2021. Recruitment resulted in 53 individuals that were interested in participation and completed the screener, of which 11 individuals did not meet inclusion criteria and 2 others could not be reached. Therefore, 40 individuals were enrolled in the study. One participant was excluded from analysis for not completing the post-intervention questionnaire on time. Some participants did not answer all questions of the post-intervention questionnaire, resulting in missing values in some analyses. The study was approved by the Ethical Committee of Antwerp University Hospital (reference number 20/26/345). All participants provided informed consent.

### Physical device for breathing exercises

**Figure 1:**
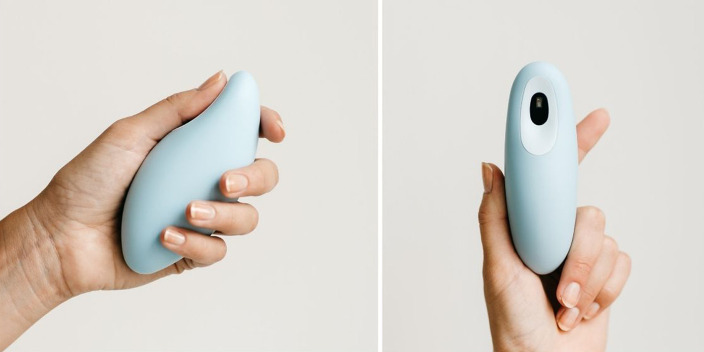
The Moonbird breath pacer, including a PPG sensor (right picture).

The current study implemented a handheld tactile breath pacer, Moonbird (Moonbird BV^1^), that incorporates a heart rate sensor and connects *via* wireless Bluetooth Low Energy (BLE) with a mobile app. The device is a tactile breath pacer, i.e., it expands and contracts in the hand, with a slow pace to which they match their breathing rhythm (Figure 1). The device measures 122 mm × 54 mm × 44 mm, has an exterior made of a biocompatible, medical-grade silicone skin. Users are instructed to hold the device in their hand and to breathe in while the device expands and breathe out while the device contracts. Heart rate data are acquired from a photoplethysmogram (PPG) sensor. The accompanying app presents real-time biofeedback. It was not the object of this study to assess the usability and impact of biofeedback. Therefore, besides mentioning the presence of this feature, no in-depth information was given to the participants.

The breath pacer can be used with a smartphone or as a standalone device, meaning that it can perform its function without needing a connection with another device. A default exercise, with adjustable duration, can be chosen in the app and downloaded on the device for standalone use. After creating an account in the app, the user can choose between individual breathing exercises or journeys. Journeys are educational audio guides based on CBT with multiple episodes per theme (e.g., less stress, better sleep and reduced anxiety). The individual breathing exercises come with (optional) auditory guidance, an adjustable duration, or a personal breathing rhythm. Another option is to let the app calculate an ideal breathing rhythm based on the users' heart rate. During the exercises, the app provides biofeedback to monitor progress regarding heart rate and HRV and indicates whether the breathing happens coherently (i.e., whether heart rate and breathing rate are in sync). Statistics are accessible afterwards. The app also includes reminders for exercises, breathing tips, a chat function, a display of the battery level, and firmware updates.

### Measures

#### The Pittsburgh Sleep Quality Index (PSQI)

The Pittsburgh Sleep Quality Index (PSQI) is a validated self-assessment questionnaire that assesses sleep quality and disturbance over a one-month period (55). The 19 items generate seven component scores with a value between 0 and 3: subjective sleep quality, sleep latency, sleep duration, habitual sleep efficiency, sleep disorders, use of sleep medication, and daytime dysfunction. The sum of the component scores compiles to a total score between 0 (no sleep problems) and 21 (severe sleep problems) (poor Cronbach's *α* of 0.584 in the current sample). A person with an overall score of 5 or more is considered as having poor sleep quality or a sleep disorder (55, 56) The scoring method for component 3 (sleep duration) had a gap in both the English and Dutch versions. The provided delineation of “>7 h; 6–7 h; 5–6 h; <5 h” contained a double counting, which is why we recalculated these categories to “≥7 h; 6–6.99 h; 5–5.99 h; ≤4.99”.

#### The Non-Restorative Sleep Scale (NRSS)

The Non-Restorative Sleep Scale is a validated self-assessment questionnaire that evaluates whether sleep is sufficiently restorative or refreshing (57). The questionnaire consists of 12 questions of which 10 items are scored on a Likert scale with values from 0 to 10. The remaining two items are scored on a 5-point Likert scale. The 12 questions evaluate four factors: refreshment through sleep, physical/medical symptoms, functioning during the day, affective symptoms. All items are given a weighted score from one to five. The total score ranges from 12 to 60 (excellent Cronbach's *α* of 0.85 in the current sample). The higher the score, the more one experiences sleep as restorative or refreshing. Persons with an overall score of 46 or less are assessed as having insufficiently restorative or refreshing sleep.

### Procedure

**Figure 2:**
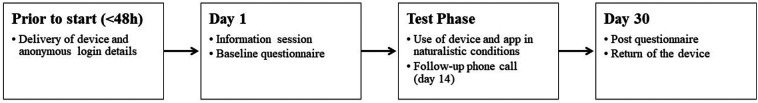
Visual representation of the procedure of the research.

Each wave of 20 participants tested the breath pacer and application for four continuous weeks. As Figure 2 shows, prior to the start of the testing period (with a maximum of 48 h before the information session), the necessary materials, including unique, pseudonymized login details were delivered. Each testing period started with an information session to explain the purpose of the research, the use of the device, and the installation of the accompanying smartphone app. Due to the Covid-19 pandemic, devices were delivered by the postal service and the information session was held online. After the online information session, participants completed the baseline questionnaire consisting of the Pittsburgh Sleep Quality Index (PSQI), the Non-Restorative Sleep Scale (NRSS), and additional questions referring to age, sex, education and smartphone use (rating their smartphone skills on a scale from 0 (totally unskillful/I can't use a smartphone) to 10 (very skillful/I can use a smartphone very well)). Participants were asked to use the device whenever experiencing the need for it (e.g., experiencing insomnia or stress) during the testing phase. A follow-up phone call (on day 14 approximately) aimed to detect any problems, provide support, and enhance compliance. On day 30, participants were asked to complete the post-intervention questionnaire consisting of the PSQI, NRSS and additional questions about the functionality, user-friendliness, and willingness to pay for the device. The testing period ended with the return of the device.

### Data analysis

For the qualitative analysis, two authors (SV and NDW) independently analyzed the open-ended questions through content analysis. For each open-ended question, the raters individually extracted one or more themes from each response. Codes and categories were generated by hand and frequencies were documented. After both raters performed this analysis, results were compared and disagreements were resolved through discussion, after which the final themes and categories were reported. Quantitative data was analyzed with IBM SPSS Statistics version 27. Descriptive statistics were used to analyze user experience data. We used a paired samples *t-*test to explore whether sleep quality and refreshment of sleep significantly improved after the use of the device for personalized breathing.

## Results

### Descriptive statistics

A total of 39 participants (26 women and 13 men) were included. Age ranged between 19 and 67 years, with a mean of 43.13 (*SD* = 12.86). All participants completed secondary education and 29 participants attained a bachelor's degree or higher. They were skilled in using a smartphone, giving themselves an average score of 8.03 (*SD* = 1.65) out of 10 on smartphone skills. None of the participants provided a score lower than 5. Most participants reported that they could install a new app easily (*N* = 11) or very easily (*N* = 26), with only 2 participants finding it difficult. Most participants used an Android smartphone (*N* = 24). All participants who completed the full PSQI had a score above 5 at baseline, indicative of problems in sleep quality (range 6–17), and the majority of the participants (*N* = 36) had an NRSS score of 46 or lower, indicative of a perception of insufficiently refreshing sleep (range 23–52). Sixteen participants were using sleep medication.

### Usability evaluation

#### The breath pacer

Participants mostly used the technology 1–6 times per week (Table 1) and could provide multiple reasons for use of the technology. The majority used it for problems with falling asleep in the evening (*N* = 27). Additional common motivations consist of training, such as breathing exercises (*N* = 18), and for problems in falling asleep after waking up at night (*N* = 13). Although the current study focused on sleep problems, participants additionally used the technology for stress reduction (*N* = 16) and, to a lesser extent, also for anxiety reduction (*N* = 4). Two individuals used it for relaxation during the day and when reading on their laptop. The technology-assisted breathing exercises were predominantly executed in the bedroom (*N* = 26), living room (*N* = 10), workplace (*N* = 2), or outdoors (*N* = 1).

**Table 1:** Self-reported frequency of use of the breath pacer and accompanying application during the testing period. Entries represent the number of individuals (*N* = 39).

Frequency of use	Breath pacer	Smartphone application
Daily	4	1
4–6 times per week	17	11
1–3 times per week	15	9
Less than one time per week	2	10
Just once	1	6
Never	0	2

Table 2 shows that most participants were favorable towards the device in terms of ease of use, registration of heart rate, and perception of breathing rate. Opinions differed regarding to what degree efforts were required to match the breathing rate with the device. The size of the device was deemed suitable for most individuals (*N* = 27), the remainder of the sample did find it somewhat (*N* = 10) or truly (*N* = 2) too large. The silicon material on the exterior was suitable for all participants. Color preferences went toward black grey (*N* = 13), sky blue (*N* = 8), blue green (*N* = 7), soft white (*N* = 3), no preference (*N* = 7) or none of the above (*N* = 1). Personalizing the device, for example with a soft cover, was of little (*N* = 13) or no added (*N* = 17) value for most of the participants. For nine participants, it had rather to a great added value. When asked about the positive aspects of the breath pacer, the design was greatly appreciated, in terms of shape (*N* = 12), material (*N* = 9) and size (*N* = 3) (Table 3).

**Table 2:** Usability statements regarding the use of the breath pacer, application, and the technology as a whole.

	Strongly disagree	Disagree	Neither agree nor disagree	Agree	Strongly agree
Use of the breath pacer					
The Moonbird device is easy to use	0	1	3	13	22
Using the Moonbird device frustrated me	18	8	3	7	2
Placing the thumb on the sensor of the Moonbird device is a pleasant way for me to determine my heart rhythm.	3	1	5	12	18
The breathing motion of the Moonbird device is easy to feel.	5	5	1	8	20
Matching my breathing to the breathing rhythm of the Moonbird device requires a lot of effort for me.	13	11	1	9	5
Use of the application					
Installing the Moonbird app on my smartphone is easy for me.	0	0	2	7	28
I think logging on to the app is too difficult.	22	10	4	1	0
It is easy for me to find what I am looking for in the Moonbird app.	1	7	7	12	10
Technology as a whole					
Using Moonbird reduces my sleep problems.	6	4	9	14	5
I find Moonbird easy to use.	0	2	1	11	23
I would recommend the use of Moonbird with the accompanying app to friends and family.	2	5	5	16	10

**Table 3:** Summary of positive aspects and important points of attention regarding the breath pacer.

Theme	Positive aspects (*N*)	Negative aspects (*N*)
Design	Good shape (12)	Size does not suit everybody (too large) (6)
	Good texture/material/feeling (9)	Uncomfortable shape (1)
	Good size (3)	Poor material (1)
	Good design (not further specified) (3)	Green light disturbs (1)
	Attractive packaging (2)	
	Light weight (1)	
Function	Helps to focus and regulate breathing (7)	The standalone function is too sensitive (4)
	Relaxing effect (7)	Default standalone exercise is too short (2)
	Movements and vibrations are relaxing (2)	It is hard to adjust breathing (1)
	After intensive use, you can use techniques without device (1)	There is a lack of feedback on the device (1)
	Determination of personal ideal breathing (1)	
	Data collection (1)	
	Combination of device and app functionalities (1)	
Usability	Easy to use (8)	Usability can be improved (1)
	Ability to use without smartphone (3)	Use with a smartphone is too much hassle (1)
Technical aspects		Perception of breathing rhythm can be improved (6)
		Communication between device and app can be improved (4)
		Noise from device can be reduced (2)
		Battery life and charging can be improved (2)
		Device lacks indicator of the battery level (1)
		Audio contains disturbing background noises (1)
		On-off switch is lacking (1)
		HR point of contact can be improved (1)
		Does not start standalone function without sufficient battery life to complete it (1)
		Standalone function does not always work (1)
Other	Good manual (1)	Lack of awareness about standalone function (1)
		*No negative aspects observed (8)*

Most positively evaluated functions were that it helped to focus and regulate breathing and that it had a relaxing effect (e.g., stress reducing or sleep inducing). It was also perceived as easy to use.

*“[Moonbird] has a stress-reducing and calming effect. The shape and texture of the device are also pleasant because they are not too conspicuous.”* (Participant 33)

Important points of attention were that the perceivability of the breathing rhythm could be improved, that the size is not suitable for everyone, and that the communication between the device and app could be improved.

*“The device did not sit well in the hand for me, which made it difficult to feel the ‘breath in and out motion’ properly; Also, the fingerprint touch sensor was not necessary for me because it required me to hold my hand in an unnatural position.”* (Participant 59)

Participants provided multiple suggestions to improve the breath pacer according to their needs (Supplementary Table S1).

The standalone function was used by 31 participants, and it worked well (*N* = 17) to very well (*N* = 13) for most of them. One participant reported that it worked very poorly. The standalone function was experienced to have somewhat (*N* = 17) to a lot of added value (*N* = 14). The remaining eight individuals (of which four did not use it) did not think it had an added value. Participants appreciated the convenience of usage independent from the app (e.g., at night), good responsivity and performance of the device, and that it offered a swift solution (e.g., in stress situations) (Supplementary Table S2).

*“[The standalone function] is easier to use than with the app and faster if you are in some kind of stress situation. Use in company of others is more inconspicuous and it is better to use at night”* (Participant 35)

The most often reported critical aspect refers to the standalone function being overly sensitive (*N* = 5), as reported by a participant:

“*It barely needs a ‘shake’. It turns on very quickly and the light often comes on without me having much to do with it*.” (Participant 47)

#### Smartphone application

Frequency of use of the application varied but was lower than the use of breath pacer, with eight participants using it just once or never at all (Table 1). The two individuals who did not use it, reported that they had an older phone and they weren't interested in smartphone use (*N* = 1) or that they did not have their smartphone nearby in the evening (*N* = 1). Participants could provide multiple reasons for use of the application, of which the most common one was to start a breathing exercise (*N* = 30). Other reasons consisted of maintaining a record of statistics such as completed exercises (*N* = 16), following journeys with additional information (*N* = 16), and setting up a personal breathing scheme or exercise (*N* = 13). The app was used much less for consulting information on the device (*N* = 4) or setting reminders for breathing exercises (*N* = 2). Other reasons were to check the battery levels of the device (*N* = 1) and to send a chat message with a question to the company (*N* = 1).

Table 2 shows that installing the app and logging into the app was easy for the majority of participants. Nevertheless, one in five participants indicated that it was not always easy to find what they were looking for in the app. Most participants reported that the smartphone application provided somewhat (*N* = 16) to a lot (*N* = 13) of added value. Eight individuals did not believe the application had a lot of added value, of which two did not find it useful at all.

When asked to elaborate on their experiences with the application, many participants refer to the added value of journeys and the availability of statistics and personal follow-up (Supplementary Table S3).

*“For me, the journeys you could follow made sure I was even more focused on Moonbird, and kept my mind from wandering to other things. Straying thoughts to other things often prevents me from being able to sleep. As long as my focus stayed with Moonbird through the spoken text, I fell asleep faster. It is also easier for me to focus on spoken text, than to focus on a quiet sound of raindrops or whales and so on, which other apps often use (think ‘Calm' app or ‘Sleep Cycle' app). Other apps also offer spoken text, but mostly in English. The Dutch language sounds much more familiar and is therefore better for me. I also liked that the spoken text actually teaches you something.”* (Participant 57)

The most frequently reported negative aspect was that participants disliked using their smartphone in the bedroom or at night. The most frequent suggestion related to solving technical issues (e.g., bugs, stability; Supplementary Tables S3, S4).

#### Individual breathing exercises

The majority of participants executed at least one breathing exercise (*N* = 36). These exercises can be combined with voice guidance, which was used by 26 participants. The remaining 10 participants indicated that they did not use the voice guide because this was distracting and they experienced use without audio to be more relaxing (*N* = 3), participants wanted to avoid smartphone use in the bedroom (*N* = 3), they used it in front of the tv so thought audio was disturbing (*N* = 1), they were not interested (*N* = 1), or they did not perceive any added value (*N* = 1). Several participants were positive about the audio, although some also did not deem the voice suitable (Supplementary Table S5). Suggestions for the breathing exercises mostly focus on the voice, for example, to use professional voice artists (e.g., ASMR-tuned voices; Supplementary Table S6).

“*The voice is clear and understandable. I would give the voice a 6.5 out of 10 for use with this app. In my opinion, there are better voices (think professional voice artists) that could provide even better results (I compare the voice to other apps like ‘Calm’).”* (Participant 57)

While starting exercises and using voice guidance was easy, adjusting the length of an exercise, adjusting the breathing pace of the device, and stopping exercises early proved difficult for one in five participants (Supplementary Table S7). Setting up a personalized exercise based on heart rate was clear to few people at the outset and there was some ambiguity regarding the visual representation of the heart rate parameters in the app. In the follow-up phone call, multiple participants received additional information on these latter functions so as to provide them the opportunity to test them by the end of the pilot.

#### Journeys

A total of 21 participants embarked on at least one journey. Other participants did not do journeys because they were not interested in using the app (*N* = 4), were not interested in the journeys (*N* = 3), did not want to use a smartphone in the bedroom (*N* = 1), were not able to connect with the app (*N* = 3), did not like the audio (and had to repeat exercises due to a bug; *N* = 1), were unaware of the journeys (*N* = 1), or due to time constraints (*N* = 1). Most of these 21 participants who completed at least one journey thought these journeys had somewhat (*N* = 13), to much (*N* = 6) added value with only two reporting limited added value. Participants generally found these journeys easy to work with and interesting (Supplementary Tables S7–S9).

#### The entire technology

Table 2 shows that about half of the sample reports some reduction in sleep problems after using the breath pacer and accompanying application. The technology does prove to be generally easy to use and the majority of participants would recommend the technology to friends and family.

### Exploratory analysis regarding changes in sleep quality and refreshment of sleep

The PSQI scores at baseline and post-test suggest that the sleep quality was significantly higher after the use of the breath pacer, as shown by a mean reduction of three points on the total scale (Table 4). Further inspection of the subscales shows improvements in self-reported subjective sleep quality, sleep latency, sleep duration, and sleep disturbances. Habitual sleep efficiency and use of sleep medication did not change significantly during the testing period. The NRSS scores at baseline and post-test indicate an improvement (of 5.49 points) in how refreshing participants perceived their sleep to be after the use of the breath pacer (Table 4). All NRSS subscales improved significantly.

**Table 4:** Scores on the Pittsburgh Sleep Quality Index (PSQI) and Non-Restorative Sleep Scale (NRSS) subscales at baseline and after the test.

	Baseline		Post-test		Change	Paired *t*-test		
	Mean	SD	Mean	SD	Mean [95% CI]	*t* value	*df*	Sig (two-tailed)
PSQI total	11.28	2.62	8.27	2.88	−3.00 [−4.07, −1.93]	5.69	35	<.001
PSQI subjective sleep quality	2.23	0.48	1.41	0.59	−0.82 [−1.05, −0.59]	7.11	38	<.001
PSQI sleep latency	2.36	0.78	1.69	0.80	−0.67 [−0.93, −0.41]	5.17	38	<.001
PSQI sleep duration	1.24	0.94	0.92	0.98	−0.29 [−0.55, −0.03]	2.22	37	.03
PSQI habitual sleep efficiency	1.19	0.88	0.97	1.04	−0.22 [−0.58, 0.14]	1.21	36	.23
PSQI sleep disturbances	1.41	0.50	1.21	0.41	−0.20 [−0.37, −0.04]	2.45	38	.02
PSQI use of sleep medication	1.13	1.40	0.92	1.33	−0.20 [−0.43, 0.02]	1.84	38	.07
PSQI daytime dysfunction	1.74	0.68	1.18	0.72	−0.56 [−0.78, −0.34]	5.18	38	<.001
NRSS total	36.05	8.21	41.54	8.29	5.49 [3.43, 7.54]	5.40	38	<.001
NRSS refreshment from sleep	7.15	2.78	9.28	2.85	2.13 [1.28, 2.98]	5.06	38	<.001
NRSS physical/medical symptoms	14.0	3.95	15.26	3.81	1.26 [0.19, 2.33]	2.38	38	.02
NRSS daytime functioning	8.67	2.79	10.26	2.89	1.59 [0.77, 2.41]	3.92	38	<.001
NRSS affective symptoms	6.23	1.99	6.74	1.74	0.51 [0.03, 0.99]	2.13	38	.04

## Discussion

The current mixed-method pilot study investigated the acceptability and usability of a handheld breathing device, accompanied by a smartphone application. Previous research shows that e-Health tools can suffer from low uptake or high discontinued usage in practice (58–60). Fundamental problems with the design (i.e., shape and function) limit the impact in practice (61). Therefore, a user-centered approach is necessary to facilitate adoption and implementation in the context of the target population. Participants of this study used a tactile breath pacer for one month, mostly multiple times per week, and results suggest adequate usability and good potential for implementation.

Overall, participants evaluated the breath pacer and supporting app positively: it helped them to focus, to regulate breathing and to relax. Features that were greatly appreciated included the standalone function, the look / feel (material and design), and the biofeedback options in the app. On the other hand, participants identified areas for improvement consisting of size adjustments, an increase of the perceptibility of the breathing rhythm of the device and a clearer interpretation and visualization of the biofeedback parameters. Participants' subjective sleep quality and refreshment from sleep improved significantly after using the device. The observed improvement of three scale points could be clinically meaningful since a Minimum Clinically Important Difference (MCID) of 3 has been proposed for the PSQI (62), although recent work has also suggested a MCID of 4.4 after a 6-month intervention (63). The NRSS also showed significant improvement, but the authors are not aware of an established MCID for the NRSS. The findings of the current non-controlled pilot study provide preliminary support for the short-term benefits of using a tactile breath pacer to improve sleep quality, however, further research is required and it is advisable that patients with severe insomnia receive additional support from healthcare professionals to guarantee clinically significant improvements.

The current study brought to light several strengths and opportunities for improvement for wearable breathing devices and other eHealth solutions. Based on these findings, recommendations can be given to developers of wearables and eHealth, shown in Table 5. However, it is important to keep in mind that the current study concerns the acceptability and usability of one specific breath pacer in a community sample and findings might not generalize to other devices or target groups.

**Table 5:** Recommendations for developers of eHealth tools and wearable breathing devices.

	Direction	Application to wearable breathing devices
1	Adapt the shape, size and material to the specific target group and the (place of) use.	Provide a tactile breath pacer in multiple sizes to account for differences in hand size and morphology.
2	Make pairing with other devices, like a smartphone, optional.	Design a wearable and standalone breath pacer to accommodate multiple purpose use.
3	Design for simplicity since users expect autonomous use.	Design the tools to be intuitive but provide a brief, yet comprehensive, manual or instruction for the breathing device.
4	Support the know-how of users that expect more guidance by adding evidence-based content	Provide sufficient (but optional) educational and evidence-based content about breathing and related subjects.
5	Inform users about their progress.	Make direct (bio)feedback available through insightful representation of users’ results.
6	Investigate the usability of the device with a user centric approach in addition to its effectiveness.	Perform usability research with the device of interest. Relatedly, offering users the opportunity to report (acute) problems, and addressing such problems quickly can prevent drop-out.

Some limitations require discussion. The current naturalistic pilot study had a limited sample size and lacked a control group. However, the sample size is satisfactory to detect usability problems and inform on design preferences (35). Additionally, a diversified age profile was included to promote representativeness of the target population. In order to measure effectiveness and causal relationships pertaining to the breathing device and its specific features, further clinical and preferably longitudinal research is needed. A recent study investigating the effect of breathing exercises *via* HRV biofeedback on sleep quality did result in PSQI scores below the cut-off of 5. However, the participants in this study were asked to train at least 100 min a week and to follow ten training sessions (64). Frequency of performing the exercises can thus play an important role here. In addition, effectiveness research should also go beyond questionnaire-based assessments of sleep quality and include objective assessment of sleep.

In conclusion, the current study observed that technology-supported breathing exercises with a tactile breath pacer are a feasible and acceptable intervention that could be an effective way to improve sleep quality. While the effectiveness of breathing exercises has a long-standing evidence base, the eventual uptake and implementation of these technology-supported exercises largely depend on the usability and acceptability for end users. The current user-centric approach, supported by living lab research, is therefore a unique way to explore the preferences and needs of users in the design process.

## Data Availability

The raw data supporting the conclusions of this article will be made available by the authors, without undue reservation.

## Footnotes

^1^ https://www.moonbird.life/how-it-works
