# Supplementary material for: Evaluation of a tactile breath pacer for sleep problems: A mixed method pilot study
Source: Front Digit Health. 2022 Oct 5;4:908159. doi: 10.3389/fdgth.2022.908159 (PMC9581241; doi:10.3389/fdgth.2022.908159)
Supplement: Supplementary file 1 [file DataSheet1.docx]

**Supplementary materials**

**Table S1.** Suggestions for the breath pacer

| **Suggestion** | **N** |
| --- | --- |
| *Function of the device* |  |
| Improve how well you can feel breathing rate | 4 |
| Add music or nature sounds to device | 2 |
| Automatically apply optimal breathing determined at intake to exercises | 1 |
| Clearly inform about standalone function | 1 |
| *Design of the device* |  |
| Adjust size of device | 3 |
| Make it look less like an adult entertainment item (change look or add logos) | 2 |
| Offer it in different (happy) colors | 2 |
| Change material to something more luxurious | 1 |
| *Technical aspects* |  |
| Facilitate switching it on and off (e.g., with switch) | 2 |
| Improve charging (efficiency or how it is charged) | 2 |
| Remove green light and reduce noise further | 1 |
| Reduce sensitivity of standalone function | 1 |
| Resolve connection issues with app^a^ | 1 |
| Better notifications of firmware updates | 1 |
| Improve contact with HR measurement point | 1 |
| *Other* |  |
| Make wrist version with wireless earbuds | 1 |
| Add cleaning product | 1 |

^a^ This participant experienced technical difficulties

**Table S2.** Results of the thematic analysis of the positive aspects and important points of attention regarding the standalone function.

| **Positive aspects** | **N** | **Negative aspects** | **N** |
| --- | --- | --- | --- |
| Convenient (more so than through app) | 8 | Overly sensitive | 5 |
| Good responsivity and performance of device | 8 | Long response time | 3 |
| Can be used quickly | 4 | Default exercise is too short | 2 |
| Prefer not to use smartphone (before sleeping) | 2 | Prefer to use the app | 2 |
| Possible to combine with listening to the radio | 1 | Was unable to switch it off | 1 |
| More discrete | 1 | Requires some experience to use it | 1 |
| The breathing motion of the device is easy to feel | 1 | Wakes up my partner | 1 |
|  |  | Experienced technical error | 1 |

**Table S3.** Results of the thematic analysis of the positive aspects and important points of attention regarding the smartphone application.

| **Positive aspects** | **N** | **Negative aspects** | **N** |
| --- | --- | --- | --- |
| The journeys provide added value | 12 | Don't like to use a smartphone in bedroom/at night | 4 |
| Statistics & personal follow-up | 9 | HRV statistics and interpretation can be improved | 2 |
| Relaxing voice | 3 | Breathing exercises from the app are distracting | 2 |
| Exercises are interesting to follow | 3 | Using the app is too much effort | 1 |
| App provides guidance and/or promotes engagement | 2 | Lacks automatic registration and data collection of standalone sessions | 1 |
| Personalisation of exercises | 2 | Need to redo exercises when leaving the app early | 1 |
| Combination of app and device is interesting | 2 | No need for exercises | 1 |
| Promotes self control | 1 | Want to reduce screen time | 1 |
| Standalone fucntion is not enough, I need app functions | 1 | Lack of personalisation | 1 |
| I needed long session with voice before I could relax | 1 | Achieving full app potential requires exercise | 1 |
| I prefer spoken word (in native language) over relaxing sounds/music in other application | 1 |  |  |

**Table S4.** Suggestions for the smartphone application

| **Suggestion** | **N** |
| --- | --- |
| Improve the app technically (e.g., stability, bugs) | 4 |
| Remove the need to complete a journey to start next one | 2 |
| Provide more information on how to interpret feedback | 2 |
| Improve the unpleasant voice | 1 |
| Avoid negative phrases instructing user "not to do" something | 1 |
| Using the app requires some training | 1 |
| The app is in need of visualitation of the data/statistics | 1 |
| Add a dark mode to the app | 1 |
| Introduce time to get comfortable before exercise starts | 1 |
| Allow to follow journey multiple times | 1 |
| Develop an accompanying web page to provide information and follow up | 1 |
| Add stories in same soothing voice | 1 |
| Provide more meditation (as opposed to information on stress and reasons for poor sleep) | 1 |

**Table S5.** Results of the thematic analysis regarding the positive aspects and important points of attention regarding the audio.

| **Positive aspects** | **N** | **Negative aspects** | **N** |
| --- | --- | --- | --- |
| Pleasant voice | 9 | Unsuitable voice (e.g., monotonous, slow) | 4 |
| Relaxing voice | 5 | Voice bothered me/induced stress | 2 |
| Voice is clear | 2 | Content was too technical and not relaxing | 2 |
| Effective | 1 | Too much use of negative phrasing with adverse effect | 1 |
| interesting | 1 | Background noise | 1 |
|  |  | Not enough variation in exercises | 1 |
|  |  | Did not like the accent of the voice | 1 |
|  |  | Sometimes the breaks between text were too large (unclear whether exercise was over or not) | 1 |

**Table S6.** Suggestions for the breathing exercises.

| **Suggestion** | **N** |
| --- | --- |
| Use professional voice artists | 3 |
| Exercises are good but require practice | 1 |
| Offer multiple voices to choose from | 1 |
| Use warm voice in Flemish-Dutch accent | 1 |
| Provide end tune/sentence so you know when exercise is done | 1 |
| Improve accuracy of breathing rate | 1 |
| Offer more meditation | 1 |

**Table S7**. Usability statements regarding the breathing exercises and journeys.

|  | **Strongly disagree** | **Disagree** | **Neither agree nor disagree** | **Agree** | **Strongly agree** |
| --- | --- | --- | --- | --- | --- |
| **Breathing exercises** |  |  |  |  |  |
| I find it easy to start a breathing exercise | 1 | 1 | 0 | 11 | 23 |
| It is clear to me how to adjust the length of an exercise | 2 | 5 | 5 | 12 | 12 |
| I find it easy to change the breathing pace of my Moonbird in the app | 2 | 5 | 3 | 17 | 9 |
| It is clear to me how to turn the audio guidance on and off during an exercise | 0 | 5 | 3 | 7 | 21 |
| I know how to stop an exercise early. | 2 | 5 | 2 | 10 | 17 |
| It is clear to me how to create my own exercise in the Moonbird app. | 5 | 10 | 8 | 8 | 5 |
| It was clear to me what the visual representation of HRV, coherence and HR in the app meant during the exercise. | 4 | 9 | 6 | 11 | 6 |
| **Journey** |  |  |  |  |  |
| I find it easy to start a journey | 0 | 0 | 1 | 7 | 13 |
| The journeys are interesting for me | 0 | 0 | 1 | 11 | 9 |
| It is clear to me which journey I have already completed and which I have not in the app. | 0 | 1 | 1 | 5 | 14 |

**Table S8.** Results of the thematic analysis regarding the positive aspects and important points of attention regarding the journeys.

| **Positive aspects** | **N** | **Negative aspects** | **N** |
| --- | --- | --- | --- |
| Interesting and informative | 9 | Exercises are enough | 1 |
| Helps to focus on breathing | 4 |  |  |
| Relaxing | 3 |  |  |
| Combination of informative journeys and with a breath pacer | 1 |  |  |
| Good for follow up | 1 |  |  |
| Nice to follow journey | 1 |  |  |

**Table S9.** Suggestions for the journeys.

| **Suggestion** | **N** |
| --- | --- |
| Apply it more to the person | 1 |
| Provide additional journeys with simple and varying stories and podcasts | 1 |
| Allow to choose which trajectories you do (instead of the mandatory chronological order and the need to complete the last one before starting a new one) | 1 |
